# Post-disaster (im)mobility aspiration and capability formation: case study of Southern California wildfire

**DOI:** 10.1007/s11111-023-00416-5

**Published:** 2023-04-11

**Authors:** Nick Tinoco

**Affiliations:** Department of Sociology, UCLA, Los Angeles, CA USA

**Keywords:** Immobility, Community Mobilization, Place attachments, Disasters

## Abstract

Scholarship on the environmental dimensions of migration demonstrates the complex interplay of climatic and non-climatic factors which combine to create a potential for migration. Yet in times of environmental crisis or change, not everyone aspires to or is capable of moving to reduce their vulnerability. When, why, and how populations vulnerable to hazard risks decide not to migrate remains a significant gap in our understanding of the migration—environment relationship. Analysis of data from 38 qualitative interviews shows how Los Angeles County residents—after surviving the 2018 Woolsey Fire—developed aspirations to stay and/or rebuild, depending on the attachments and meanings associated with their communities. This paper also seeks to clarify the concept of capabilities to stay by considering separately the capabilities to return and rebuild from the capabilities to cultivate preparedness. Many who stayed also worked to strengthen community resilience to alleviate concerns of future wildfire risk. Some residents expressed individual commitments to stay and defend homes during future fires, while well-equipped volunteer fire brigades have proliferated in more affluent areas. Community mobilizations pressured local government and fire services to address the perceived institutional failure during previous fire responses and fostered feelings of collective efficacy among residents which increased their confidence to remain in high wildfire risk communities.

## Introduction

This study investigates why and how disaster-affected residents stay put in high hazard-risk areas. At a time when climate change is understood to be exacerbating disaster risk, it draws on a case study of residential decision-making after the 2018 Southern California Woolsey Fire (referred to as Woolsey from here on) which destroyed 1075 mostly middle- and upper-class residential homes in Los Angeles and Ventura counties. Despite escalating risks for larger, more frequent and severe wildfires in California (Goss et al., 2020; Iglesias et al., 2022; Williams et al., 2019), and the traumatic disruptions wrought by the fire on thousands of residents, most Woolsey survivors aspired to stay and rebuild lost homes rather than relocate to lower-risk areas.

That disaster survivors tend to rebuild, as disaster research has consistently suggested, highlights the varied and complex nature of the migration-environment relationship (Bukvic & Owen, 2017; Greer et al., 2020; Young, 2018). Environmental migration scholarship is principally concerned with unpacking the multi-causal interplay of climatic and non-climatic factors that combine to produce a likelihood for migratory outcomes (McLeman, 2013; Hunter et al., 2015; McLeman & Gemenne, 2018). Yet while migration is one possible outcome when people’s security is threatened by environmental disruptions, people might also lack either the desire or the ability to migrate, meaning that the same combination of socio-economic, political, and environmental forces can simultaneously produce divergent mobility outcomes where some choose (or are forced) to leave while others choose (or are forced) to stay put (Zickgraf, 2018). To address this paradox, recent scholarship has stressed the need for developing a “mixed migration paradigm” that unifies migration drivers and constraints along with individual-level motivations to leave or stay in order to better capture the wide spectrum of outcomes separating mobility decisions that are forced from those that are voluntary (Schewel, 2021). Such a framework is also well equipped to address under-studied questions of why and how populations voluntarily opt to stay in contexts of environmental crisis (Adams, 2016; Zickgraf, 2021).

In this context, this paper asks two core questions: First, how were (im)mobility aspirations formed by Woolsey survivors and what factors made the development of migration or immobility aspirations more or less likely? Second, how did Woolsey survivors stay? What did the staying process require of returning residents and (how) did they adjust their lives to strengthen their capabilities to remain in place alongside escalating wildfire risk? Following a review of relevant theories and literature along with a description of the research site and methodology, this paper analyzes interview and archival data to address these questions. Survivors described an array of personal attachments to their communities, through connections with family or neighbors and attachments to the natural landscape, along with other personal obligations which informed widespread aspirations to stay and rebuild. However, financial constraints, particularly issues of un- or under-insurance, sometimes delayed planned rebuilds or compelled would-be rebuilders to abandon their plans and sell their burned-out lots. For returning residents, recovery activities often extended far beyond replacing lost homes; many are continuing to actively mobilize with neighbors to build community resilience and cultivate feelings of collective efficacy after confidence in local government and fire services declined following the perceived institutional failure in the Woolsey response. Residents articulated a need to take matters into their own hands and strengthen community self-reliance through organizing well-equipped volunteer fire brigades and developing strategic action plans to stay behind and defend homes themselves during future fires. Thus, this study contributes to the emerging literature on voluntary non-migration in the context of environmental crises by exploring the financial and social resources that rebuilding residents draw upon to realize their aspirations to stay (i.e., their capabilities to stay) and the ongoing, often collective process through which these capabilities are developed.

## Background

The emerging literature placing immobility in an environmental context draws heavily on the aspiration-capability framework of migration decision-making to examine how exposure to environmental disruptions influence mobility aspirations and outcomes (Zickgraf, 2018). This framework distinguishes migration aspirations from the material and social resources that endow prospective migrants with the capability to relocate (Carling, 2002; Carling & Schewel, 2018; de Haas, 2021). This allows for different categories of both voluntary and involuntary immobility to be included alongside migratory outcomes and studied jointly in the same model. As such, this framework is well suited for studying the climate-migration relationship as it enables researchers to uncover the gradual effect of climatic factors on migration decision-making processes over time as prospective migrants continually renegotiate the positive and negative elements of their place of residence alongside their capabilities to move (Van Praag & Timmerman, 2019).

However, environmental immobility scholarship has thus far primarily analyzed the sources of involuntary immobility or trapped populations: those who “not only aspire but also need to move for their own protection but who nevertheless lack the ability” (Black & Collyer, 2014, p. 54). In some cases, environmental change decreases out-migration by reducing migration capabilities, particularly if a community’s economic livelihood is based on agricultural production or resource extraction (Foresight, 2011; Nawrotzki & Bakhtsiyarava, 2017). Evidence of this “holding” effect of place has been observed in countries such as Burkina Faso (Gray & Wise, 2016) and Zambia (Nawrotzki & DeWaard, 2018), while similar patterns have been identified after disasters like Hurricane Katrina (Masquelier, 2006). In contrast, the sources of voluntary non-migration in contexts of environmental crisis have received less attention. Immobility research has, according to Adams, largely “focused on financial barriers to migration, and not socio-psychological or affective aspects of the decision to migrate” (Adams, 2016, p. 438). Numerous cultural or personal factors can override environmental drivers of migration and encourage the formation of active aspirations to stay and adapt to changing landscapes, including place attachments, family ties, and the development of meaningful place-specific identities (Adger et al., 2011, 2013; Greer et al., 2020).

Scholars identify place attachments, or the “positively experienced bonds, sometimes occurring without awareness, that are developed over time from the behavioral, affective and cognitive ties between individuals and/or groups and their socio-physical environment,” as a key subjective construct informing aspirations to stay (Brown & Perkins, 1992, p. 284). Adams’ study of voluntary immobility in high migrant-sending communities in the Peruvian highlands reveals that the non-economic aspects of the migration decision-making process, namely, strong senses of place attachment and feelings of obligation to family, motivated decisions to stay in communities where livelihoods are increasingly threatened by environmental change (Adams, 2016). Other scholars have examined voluntary non-migration in the context of the existential threat of sea-level rise in the Pacific where indigenous pacific-islander communities resist relocation efforts based on an array of cultural, spiritual, and familial attachment to the land (Farbotko & McMichael, 2019; Kelman et al., 2019; McMichael et al., 2021; Mortreux & Barnett, 2009; Oakes, 2019). In addition, access to place-specific amenities, particularly those associated with a high quality of life, can reinforce aspirations to stay. Rachel Kimbro’s ethnographic study of mothers in Bayou Oaks, Texas, a community with a history of severe flooding, reveals that mothers chose to stay, repairing or rebuilding flood damaged homes, to maintain an ideal, “curated” environment for their children which they deemed critical for their children’s future success (Kimbro, 2022). Disruptions to place attachments often prove detrimental to survivors well-being as they are forced to reckon not only with material losses, but a loss of their “sense of place” as well—a loss which can influence post-disaster return decisions among survivors seeking to re-establish their sense of security, wellbeing, and contentment (Chamlee-Wright & Storr, 2009). Czaika and Reinprecht identify place attachments as a key decision-relevant factor informing the cognitive processes potential migrants undergo when weighing possible (im)mobility options, arguing that these attachments often bias decision-making heuristics towards preserving the status quo (Czaika & Reinprecht, 2022).

For voluntary non-migration aspirations to be realized in contexts of environmental crisis, returning residents must also possess the capability to stay; they must have the “realistic option to achieve their life aspirations where they are” (Schewel, 2021, p. 196). Scholars have outlined how disasters can exacerbate or reinforce inequalities as household-level recovery options (i.e., the capabilities of survivors to return and rebuild) are heavily shaped by both pre-existing economic resources along with social and network capital (Barnshaw & Trainor, 2010; Elliott & Pais, 2006; Howell & Elliott, 2019). The collective nature of the capability to stay must be emphasized—community associations formed among recovering residents can accrue social capital through forming horizontal linkages between community networks and governance structures. These linkages are critical in building local resiliency by enabling residents to more easily navigate local and state bureaucracies to access funding and expertise that can be mobilized to strengthen local adaptive capacity (Adger, 2003; Aldrich & Meyer, 2015).

Whereas aspirations to migrate or stay can be viewed in an inverse relationship, the social and financial resources that make one capable to stay in environmentally risky areas differ from those that would make one capable to leave. Instead, the capability to stay in this context presupposes some degree of adaptation to strengthen local capacity to absorb and manage future shocks (Carmin et al., 2015). Scholars have typically explored the relationship between climate adaptation and migration through two general frameworks: migration-as-adaptation, whereby permanent relocation reduces one’s exposure to climate risk, or migration-for-adaptation in which migration enables households to diversify livelihoods to better absorb climate shocks by strengthening social resilience through the transfer of financial resources (Scheffran et al., 2012). While numerous empirical cases illustrate the process of migration-for adaptation in action (Mohapatra et al., 2012; Musah-Surugu et al., 2017), less work has examined the process through which those who stay behind act collectively to mobilize local social and human capital for community-based adaptation projects to strengthen collective capabilities to stay, or how the outcomes of these projects might influence or reshape residents’ (im)mobility aspirations.

In sum, by largely focusing on mobility constraints rather than the factors that “retain” populations in vulnerable areas or “repel” them from potential lower-risk destinations, the environmental immobility literature still largely reinforces assumptions that in times of environmental crisis, people will want to and try to move (Schewel, 2020). To better center voluntary non-migration in these established frameworks requires, as Mallick and Schanze note, distinguishing aspirations and capabilities to *stay* from aspirations and capabilities to *move* (Mallick & Schanze, 2020, p. 4). Consequently, this paper contends that the choice to rebuild a home lost to wildfire represents more than a straight-forward, individual-level cost–benefit calculation to secure housing through the most financially viable recovery pathway. Moreover, that decision is an active, engaged, and dynamic process whereby residents process loss and trauma, reconstruct disrupted ties, and mobilize collectively to build adaptive capacity—all key factors that must be addressed for residents to feel safe and secure in their decision to stay. Thus, this paper aims to contribute to this growing literature by examining not just why people stay, but how they stay: what actions and processes do residents undertake to deliberately construct and strengthen their capabilities to stay in communities facing mounting hazard risks.

## Setting and case selection

The Woolsey Fire ignited on the afternoon of November 8, 2018, in Woolsey Canyon in the Santa Susana Mountains, near the border of Los Angeles and Ventura counties. Early the next morning, driven by strong Santa Ana winds, the fire jumped the 101 freeway and began a fast, destructive march south towards the Pacific Ocean. The 14-mile fire front, the largest and most destructive in Los Angeles County history, reached the coast by the end of the day, leaving in its wake the destruction of 1643 structures (including 1075 residential homes), three deaths, and the forced evacuation of 250,000 residents (County of Los Angeles, 2019, p. 4).

The region through which Woolsey burned has a long history of wildland fire. The topography of the mountains and canyons, the force of the Santa Ana winds which whip through the region every autumn, and the ample vegetal fuel make the greater Malibu area, in the words of Mike Davis, “the wildfire capital of North America” (Davis, 1995, p. 3). The region lost more than 2000 homes to wildfire in the second half of the twentieth century. As a result, long-time residents have, through experience, cultivated substantial knowledge of how to best prepare and harden their homes or even stay and defend their properties if necessary.

**Fig. 1:**
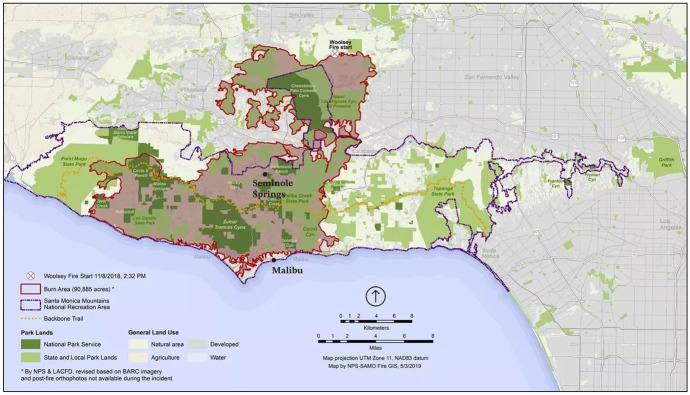
Woolsey Perimeter Map, identifies location of Malibu and Seminole Springs (National Park Service, 2019; research sites added by author)

To gain insight into the post-wildfire decision-making of affected residents, I selected two of the most severely impacted communities to be the focus of my fieldwork: Malibu and Seminole Springs. Figure 1 situates both research sites in the broader region, showing their location in relation to the Woolsey Fire burn area perimeter. In Malibu, Woolsey destroyed 488 homes (Parfrey et al., 2021, p. 12). According to the city’s official rebuilding statistics, 352 rebuilding plans have been approved by the city as of early 2023, though not all have received final permitting or began construction. In Seminole Springs— a mobile home park in the Santa Monica mountains—110 of the park’s 215 homes were lost. Residents began returning in new mobile homes in Spring 2020, roughly a year and a half after the fire. While official rebuilding statistics for Seminole Springs are not publicly available, a review of satellite imagery from October 2022 finds 20 empty burn-out lots remaining, suggesting that approximately 80% of the 110 lost homes have been rebuilt.

Four years out from the fire, many residents are yet to complete their rebuilds. Of the 352 rebuilding plans approved by the City of Malibu, only 122 have been completed. In Seminole Springs, the rebuilding process has unfolded faster, as prefabricated mobile homes are quicker and cheaper to construct, and do not require residents to contract architects to draw up unique plans for individual homes. However, damage to park utilities requiring upwards of $10 million in repairs precluded anyone from rebuilding in the first year following the fire (Sharp, 2019).

The population of Malibu and the surrounding areas tends to be highly educated and composed of many high-income earners. Malibu’s population is approximately 80% white and boasts a median household income of $178,594 in 2021, more than double the state average, (US Census Bureau, 2021). Comparable data for Seminole Springs is difficult to approximate as the small, unincorporated community is tucked away in a generally affluent region. The closest approximations available through census data are likely distorted as the tract containing Seminole Springs also includes nearby highly wealthy enclaves like Malibou Lake. Seminole Springs residents interviewed tended to identify themselves as middle-class and emphasized the “relative affordability” of Seminole Springs when compared to neighboring communities. Most residents recognized un- and under-insurance as an issue in their community. While Seminole Springs is an outlier among the generally affluent communities impacted by Woolsey, many insights can be gained from its inclusion in the sample considering the limited attention given to mobile home parks in disaster recovery research (Rumbach et al., 2020).

## Methods

Qualitative data for this study were collected via two primary means: through semi-structured in-depth interviews with Woolsey Fire survivors and a review of archived recordings of post-disaster city council meetings and information sessions held by recovery-focused non-profits and the city of Malibu. Semi-structured interviews were designed to cover five core dimensions: (i) residents’ background and pre-Woolsey wildfire risk perceptions, (ii) the process of post-Woolsey recovery aspiration formation, (iii) residents’ capabilities to realize their recovery aspirations, (iv) how Woolsey changed their perception of wildfire risk, and (v) residents’ participation in community mobilizations and views of stay and defend strategies. Interviews were conducted between February and September 2021. Due to the COVID-19 pandemic, 35 of the interviews were conducted virtually over zoom. The three remaining interviews, conducted across the final month of the data collection period, were carried out in-person at the participants’ properties. Participants would walk me through their property which afforded the opportunity to collect supplemental observations of the research sites.

Interview participants were recruited through two primary strategies; first, I reached out to several Malibu homeowner’s associations in hard-hit neighborhoods and the Seminole Springs front office to disseminate information about the study to their resident lists. Second, connections made with two organizations supporting long-term recovery—the Malibu Foundation and the Los Angeles Region Community Recovery Organization—facilitated the recruitment of additional participants. The initial sample obtained through these two means was extended through snowball sampling, as I worked to recruit through the social and community networks within the sites. My positionality as a local graduate student at UCLA, a university which many participants expressed a connection to, and my own background as a lifelong resident of the nearby San Fernando Valley who has witnessed many wildfires in the region over the years, certainly helped to recruit and establish understanding with participants.

Table 1 describes the study’s sample. In total, 38 interviews were conducted with 31 Woolsey survivors (10 from Seminole Springs and 21 from Malibu) and seven relevant experts including disaster case managers and long-term recovery organizers. Of the 31 survivors, 21 lost their homes, though others reported major smoke damage requiring significant renovation. Fourteen who lost homes decided to rebuild, and seven did not. In total, nine of the 31 residents permanently relocated after Woolsey and seven locally in Woolsey-affected areas. Interviews were audio recorded and transcribed before the data was analyzed inductively, through multiple rounds of open coding in accordance to a grounded theory approach as is commonly utilized in qualitative disaster research (Phillips, 2014). As most participants requested anonymity, pseudonyms are used throughout this paper. This project had IRB human ethics clearance through the University of California, Los Angeles (ID# 21–000542).

**Table 1:** Description of sample

	Total sample	Lost homes	Rebuilt homes
Malibu	21	13	7
Seminole Springs	10	8	7

Supplemental data were collected through the review of archived recordings of post-disaster information sessions held by recovery-focused non-profits and the city of Malibu. Recordings of post-disaster Malibu City Council meetings were reviewed at one-, three-, six-, and twelve-month intervals after the fire. Additionally, recordings of special information workshops on rebuilding, accessing insurance, and navigating the permitting process were also analyzed.

## Results

### Deciding to rebuild: why residents aspired to stay

The unprecedented destruction that Woolsey brought to the region heightened residents’ concerns of wildfire risk. While many, particularly longtime residents, have experienced wildfires in the past and consider themselves well-aware of the risk, some reported feeling “naïve” in assuming that Woolsey would not be much different than the fires they have come to expect in the region. “I’ve been through a bunch of fires in Malibu, but most of the time they didn’t directly affect me” remembers Jack, a lifelong Malibu resident whose family wants to rebuild their Point Dume home. “It was always kind of a joke, like, oh there’s another fire… [shrugs] ehh it’s not going to come here. And you kind of get desensitized to it.” Residents no longer feel desensitized to wildfire and are highly perceptive to even minute changes in weather patterns that heighten fire risk, which often proves a source of great anxiety and stress. “I am scared every time the wind is a little stronger, I am afraid a fire will break out. I never liked wind, but now I despise it,” says Michelle, a 16-year resident of Seminole Springs who rebuilt.

While several respondents alluded to neighbors who did not return explicitly out of fire-related trauma and fears of future fire risk, all interviewees in this sample explicitly expressed having an initial aspiration to stay and/or rebuild in their communities—though not all did. While such fears are common and multiple residents reported developing PTSD or other stress disorders after Woolsey, among no interviewee did fear outweigh the perceived benefits of staying, though residents often found this difficult to articulate. “I don’t know if that’ll ever go away,” said Seminole Springs resident Rosa of the traumatic emotional toll of the fire and the fears and anxieties it produced, “[but] we’re not leaving. I don’t know why. But it just, no, I’m not leaving my home. I’m not leaving this area. I love this area. This is where I want to be.”

The decision to return and rebuild a home lost to wildfire is intimately related to the process by which survivors perceive the prospects for future wildfire risk. Risk perceptions within a population can vary significantly and often contradict expert assessments. As social psychologists have noted, people tend to evaluate the potential for risk through—often subconscious—cognitive heuristics, or simplifying mental shortcuts, employed to intuitively process available information and risk signals (Czaika & Reinprecht, 2022). The most common decision heuristic observed among rebuilders mirrors the representativeness heuristic first introduced by Kahneman and Tversky, where peoples’ perception of future fire risk was articulated through direct comparisons to other hazards perceived as similar in other regions (Kahneman & Tversky, 1972). “I mean, if you live in California, there are inherent risks,” says Lynn from Seminole Springs. “Anywhere you live, if I lived in Oklahoma, I would have to be aware of tornadoes. And if I lived on the East Coast, I’d have to be aware that hurricanes can hit at any moment. So, I think it’s part of the tradeoff of living in California.” Many residents said wildfire just “comes with the turf,” and if they lived elsewhere, they would have to deal with any number of other hazards. “You pick your poison,” says Quinn, a lifelong Malibu resident who is rebuilding her home in Malibou Lake. “[Other places] you get tornadoes, you get hurricanes, you get floods.” Even if one possesses the capabilities to relocate elsewhere to reduce their exposure to wildfire risk, they would still inevitably be vulnerable to some other hazard. Any perceived wildfire risk reduction benefit does not out-weigh the transition costs of moving and the “quality of life” losses associated with leaving their preferred community.

Researchers have found decision-making heuristics to often be biased by individual, cognitive pre-dispositions that orient decision-making processes towards preserving the status quo (Czaika & Reinprecht, 2022). People tend to value what they currently have or previously had higher than what they might have, what Czaika and Reinprecht term the “endowment effect,” which can place limitations on the recovery options perceived as desirable. “[Rebuilding] is our only option to live somewhere *like this*,” Quinn continues. “Our choice from day one was to stick to the rebuild, even with how hard it is, because you can’t buy anything in L.A. for our budget, a little condo maybe, but that wouldn’t be, like, around *here*.” Living “here” in a place “like this” suggests that a desire for continuity, familiarity, and the comfort and security of home are powerful forces that continue to retain people in place despite rising wildfire risk.

Feelings of love towards one’s community and local environment were nearly universal across interviews, reflecting the salience of deeply held place-specific attachments to both the social and natural worlds residents inhabit in the formation of post-disaster recovery aspirations. Table 2 provides representative quotes that illustrate the most articulated dimensions of place and community attachments that motivated respondents to aspire to return and/or rebuild their homes. Living close to the beach and surrounded by countless coastal mountain trails, nearly all residents interviewed found that the natural beauty of the area enriched their lives. Others spoke of the strength of social attachments to nearby friends and neighbors motivating their return, along with feelings of obligations to family, such as the need to keep their kids in the same schools so as not to disrupt their social lives. Thus, Woolsey not only wrought significant material and financial losses for survivors, but it also disrupted these long-standing attachments by forcibly separating people from their neighborhoods, to the detriment of residents’ well-being.

**Table 2:** Representative quotes of reasons for staying/rebuilding

Reason for staying	Quotes
Place attachments	I was really, really involved in my life here… **my life revolved around this community**. And that's, I think, one of the reasons why [the fire] was so difficult, not only did I lose my house and all my belongings… it was a loss of my lifestyle. **I identified so heavily with my position in the community**… [After Woolsey] I kind of felt inside that I lost my identity. (Susan, 18-year Seminole Springs resident)
	It’s really the **community** and everything around you, the **memories** you have with that area, that really **makes it a home**. Even without the house being on that property, it’s still home, it’s just the house isn’t there. And yeah, [rebuilding] it’s a heavy lift, but starting a new home in a new community, that's a much, much heavier lift. (Jack, lifelong Point Dume resident)
	No, I’m not leaving my home. I’m not leaving this area. **I love this area**. This is where I want to be. I’m so close to the ocean, that’s my favorite place to be. Why would I retire and go someplace else when **I got what I want right here**? (Michelle, 16-year Seminole Springs resident)
Family and work obligations	We need to get the kids back in school as quickly as possible… I need to take care of my family and **I need to take care of my kids**. They need to have their lunches and they need to go to school and trying to have some sort of consistency for them was huge. (Stephanie, 9-year Malibu Park resident)
	I had to do family things, **I was in business consulting at the time, and I was on active projects**. So, like, what I had to do for work, and then what we had to do in terms of the home… it was helpful just having traction, for me action was a good thing mentally (Jen, 9-year Malibu resident)
	I think because we are a pretty **close family**. And because we’re all here in the area, I don’t think it occurred to any of us to give up the property. I think the immediate reaction of the family was we’re going to make this work. We’re going to rebuild. (Miranda, 51-year Malibu Park resident)

Time proved a key compounding factor; survivors with longer residency in a particular community tended to draw upon their deep history to explain their desires to return and/or rebuild. Time in place also enables residents to develop local knowledge and experience in fire safety and preparedness which increases confidence in decisions to stay. Jack, like many longtime Malibu residents, distinguishes between an “old” Malibu composed of multi-generational families with deep regional knowledge from a “new” Malibu made up of more recent arrivals attracted by the pursuit of an idyllic, “rural” life on the coast. According to Jack, who himself comes from an “old” Malibu family, “a lot of the ‘old’ Malibu people that have been here for generations, they’ve been through several fires, and there was always an understanding that in a major fire, you had to look after your own shit, and often this concept of self-reliance gets lost with affluence,” a pattern which Jack associates with “new” Malibu residents who “don’t always want to take responsibility for their decision to live in a fireplace.”

### Defining capabilities to stay: who rebuilds and who moves on

While aspirations to stay and rebuild are widespread, not all possess the capabilities to realize them. In fact, of the seven interviewees who lost a home and did not rebuild, all articulated at least an initial preference to stay and rebuild on the same property had conditions been favorable. Moreover, five of the seven ultimately decided to relocate permanently within the region, meaning that their moves did not reduce their vulnerability to future wildfire risk. That these residents were ultimately compelled to leave against their wishes, due to non-climatic financial and social pressures, and resettled locally on land with the same wildfire risk profile speaks to the fluid nature of post-disaster (im)mobility aspirations. The strength of aspirations to stay can be eroded as time passes with little progress made in obtaining permitting, to say nothing of breaking ground on the rebuild itself. Moreover, should a rebuilder find their budget sufficiently constrained, priorities might shift away from rebuilding and towards maintaining one’s financial security, even if that means selling one’s property and moving on.

Table 3 outlines representative quotes of the four most common reasons for not rebuilding (i.e., the greatest constraints on the capability of people to stay and rebuild after Woolsey): financial constraints, insurance, age, and community change. First and foremost, the steep and compounding costs of the entire process makes it unattainable for some, particularly un- or under-insured residents. Many residents describe how quickly expenses grow—contractors might raise previously established bids, or plans might need to be altered to adjust to new regulations or complaints from neighbors. Then, as one rebuilder remembers, “COVID hits and prices go through the roof because manufacturers can’t keep up with demand … We’re seeing that now building materials are 150–200% over where they were a year ago.”

**Table 3:** Representative quotes of reasons for leaving/relocating

Reasons for leaving	Quotes
Financial constraints on planned rebuild	[I thought] **of course I’m going to rebuild**, I mean there was no question at all, I was going to rebuild… and then the contractor came in with this massive, massive [bid] to rebuild which was dramatically higher than my insurance… And so, having gone through all that **battle with the costs coming in, so exorbitantly**, I took my insurance money and a year ago I ended up buying another place [in Malibu] and I’m right now in the process of trying to sell my lot. (Richard, 11-year Malibu resident)
	[We] had finally gotten permitting, and things were driving forward. All the plumbing and all the trench work was done, and we were ready to then start doing the foundation. And due to some complaints from a particular neighbor, [we] had to rip all of that up. And at that point, it’s like, **[we’re] just running out of money. We can't do this anymore**. (Stephanie, 9-year Malibu Park resident)
Insurance	We just couldn’t have built a property that could be insured in that area. We decided that it was better off just cutting our losses and moving on. If there were financing vehicles for rebuilding for the state of California, that would have been something we'd look at. But **the hurdles of financing insurance just made it impossible**. (Scott, 3-year Malibu resident)
Age	If I was 40 or 50 years old, I would go a different route [and rebuild], but I’m 70. **I don’t want to spend the next 10 or 15 years battling with this mess.** I don't want to spend the last, I mean I’ve had a really good life and I don’t want it to end like this. So, I’ve started looking at [homes in] different areas. (Elaine, 22-year Malibou Lake resident)
Community change	We had some issues [with neighbors] before the fire, but after the fire it was magnified… **it was a divisive place**, and I participated in the division, and I encouraged some antagonistic behavior, so I’m also guilty. (Percy, 29-year Seminole Springs resident)
	The camaraderie, the joie de vivre of the community, radically changed because people are forever changed because of the fires… I loved Seminole Springs, and I assumed I would live there all my life, but **it just became too hard to live with people who were so upset and angry**. (Lynn, 18-year Seminole Springs resident)

Moreover, insuring a completed rebuild is prohibitively expensive or altogether unavailable to many. Between 2017 and 2018, the California Department of Insurance reported that insurer-initiated homeowner policy non-renewals increased by 6% statewide—or 10% in wildfire-prone zip codes. While moratoriums on cancellations and non-renewals temporarily protected thousands of survivors, many residents not dropped by their insurers have seen premiums rise after insurers filed more than 220 rate increases with the state during that period. “I might consider self-insuring and literally just taking my chances on the place burning down again, because I don’t think it’s gonna burn down with what I’ve done to mitigate for fires” says Barry, who hopes that his investments in “hardening” his rebuild—the term used to describe the process of preparing one’s home to withstand wildfire—by installing two new underground water tanks storing more than 20,000 gallons, offer greater protection in the long run.

Additionally, the amount of time and energy required to navigate the rebuilding process was enough to dissuade some, particularly older residents and retirees, from rebuilding. The rebuilding process after wildfire is long, arduous, and expensive. Before receiving approval for final building permits, residents must clear debris, file insurance claims, obtain zoning approval, conduct a building safety review, compile geology/geotechnical reports, and complete an environmental health review of the site. At the same time, residents meet with architects to design their rebuild and ensure that the plans are in accordance with California’s Wildland-Urban Interface Fire Area Building Codes. Moreover, residents expressed concerns over whether—after so much time, money, and energy—the community to which they return would be the same. Some residents cited the developments of conflicts with neighbors over construction plans as a major source of frustration during the process. Others mourned community change, particularly if close friends and neighbors did not return and rebuild.

Importantly, while financial and material resources are the principal determinant of one’s capability to stay and rebuild, several residents alluded to emotional strength and the ability to process and overcome the trauma of Woolsey as an essential factor enabling them to stay. When Stephanie decided to move into Seminole Springs after plans for her family’s rebuild in Malibu Park fell through, she received many questions from concerned friends asking why she would move into an area that suffered such severe losses to Woolsey. “I guess once you lose everything, the idea of losing it again isn’t as traumatic because you know that you’re going to be able to get through it,” she responded. “Once you go through that, it’s almost like the scar tissue is stronger than what was there before.” While affect-based risk assessments emanating from direct personal experience often are based on emotions of fear or dread, feelings of confidence from overcoming past loss or of support from family, friends, and neighbors have the potential to increase self-perceptions of one’s capability to cope with and live alongside risk.

### Stay and defend: mobilizing to strengthen capabilities to stay

Early in the morning of Saturday, November 9, 2018, after the initial fire front had passed through his neighborhood in Point Dume, Jack returned with a group of friends to protect homes that were still standing from remaining spot fires. “You hear all this stuff, that turns out to be erroneous, but it sounds like Armageddon. It sounds like there's a tidal wave of flames destroying your community and, because you’re not seeing it firsthand, you paint the worst picture.” With only a basic understanding of firefighting, Jack and a group of friends quickly formed an impromptu fire brigade and began patrolling the streets and gullies they have known since childhood with shovels, garden hoses, and two-way radios. One friend, a former Marine, found higher ground and acted as a lookout, directing the group to remaining spot and ember fires over the group’s radio system, sleeping in only 15-min increments for several days. Jack’s group, which came to be known as the Point Dume Bombers in the media, adopting the name of a historic local surf crew, along with other residents who stayed behind in surrounding neighborhoods, is credited with saving dozens of homes from the fire.

Of the 31 survivors interviewed, seven stayed behind to fight the Woolsey Fire themselves, disregarding mandatory evacuation orders. “I guess I needed to know what was really happening,” says Cliff, who stayed behind in Malibu West. “You kinda realize this is a threat to us and our neighbors so I felt I should go help out.” Residents who stayed behind to defend homes exhibited several consistent factors. Most articulated the importance of self-reliance, a belief in the effectiveness of their actions based on prior preparation, along with feelings of personal responsibility to protect one’s property. “LA County just doesn't have the resources to protect everybody,” says Christian, a founding member of the Malibu West Volunteer Fire Brigade “and when [a fire] starts burning. I’m not going to stand there with my hands in my pockets and not do anything to protect our neighborhoods.”

The success of those who stayed behind during the Woolsey Fire in saving homes demonstrates that in non-extreme conditions (after the main firestorm has passed) and with the proper training and equipment, stay and defend strategies can be effective in protecting homes. As stories of residents saving dozens of homes from Woolsey spread, many residents who previously evacuated began reconsidering their prior decisions and expressed commitments to stay and defend their homes in the future. “Next time we will stay,” one resident who evacuated told the Malibu city council a month after the fire. “I don’t believe we can count on these public entities to support us [applause]… I am going to stay next time to protect my house.” “If you think a lot of people stayed back this time, next time it will be triple or quadruple, I guarantee you,” another concurred. “We will all stay and defend ourselves.” Jack, who has been working since Woolsey to turn his group into a well-equipped and well-trained fire brigade in Point Dume, believes that community engagement and direct action by residents, not the fire department, are fundamental for his neighborhood to survive future fires. “It’s just mind blowing that there’s this continued line, that first responding agencies toe that perpetuates this idea that they can always be there, because they can’t,” Jack says. “I think this concept that we can always rely on other people to save our asses, whether it’s LA County Fire, the sheriff’s department… this idea that we can rely on other people to do shit for us, it’s just the demise of society, to think that we can just rely on others to figure [it] out for us.”

In the four years since the Woolsey Fire, membership in volunteer fire brigades has grown, and new brigades have proliferated throughout the area. Christian, a 32-year resident and founding member of the Malibu West brigade, says that membership has more than doubled to 24 trained members, with more residents interested in joining, as of the summer of 2021. In Malibu West, the neighborhood’s homeowners’ association allocated $50,000 to fund the brigade, which was used to purchase equipment including turnout coats, goggles, gloves, and breathing masks along with 900 feet of professional-grade firehoses which can be attached to the community’s 27 fire hydrants or to pumps that can be fixed to the neighborhood’s 32 swimming pools. The brigade’s leadership has met with local fire chiefs, and most members have participated in at least six training days where they receive equipment instruction and strategize action plans to be employed in case of a fire before debriefing at a neighborhood barbecue. “The fact that we’re more prepared and continue to re-train, it’s really let our neighborhood be more confident and I would say more relaxed and at ease,” says Christian. “I think we’re way better off and I’m really glad that so many people have stepped up.” New brigades have emerged, often with homeowner association support, in other nearby, affluent communities.

Under increasing pressure from community groups like those organized by Christian and Jack, some city officials in Malibu have begun accepting that residents can play a role in fire suppression. They hope that fire department collaboration with well-trained stay-behind resident brigades can repair trust in the city and local fire services and dissuade residents with little training or firefighting knowledge to stay behind during an evacuation. “I know hundreds of people who say they are going to stay no matter what and that scares me.” says a Malibu city councilmember. “That’s not how you act in a fire, you really have to know what you’re doing… I think there are compromises we can come up with including local fire brigades, training, and obviously better communication with the fire command.”

The councilman is part of a group working with LA County Fire to develop a pilot program to allow certain designated, trained volunteer resident groups to stay behind and engage in fire-front following work. This means that permitted groups would shelter in safe, temporary refuge areas while the main fire-front passes, before being allowed into their neighborhoods to put out remaining spot and ember fires that follow the fire-front, which are responsible for much of the property damage in wildland-urban interface communities. This pilot program proposal would bring six resident brigades under the command of LA County Fire, allow for further training opportunities, and improve communication between brigades and fire services. While this pilot program is still in development and has not yet received final approval from LA County Fire, the organizers are optimistic that it can and will be implemented. “My hope is that if brigades from those neighborhoods show up, other residents will feel more comfortable [evacuating],” the councilman says, “and I know not everyone will evacuate, but the simple fact is staying behind with a garden hose, it’s not really a plan, it’s a prescription for disaster.”

It is important to note that comparably equipped brigades have not yet been formed in Seminole Springs. Seminole Springs residents described the community’s financial situation as weakened following the steep costs of repairing park utilities and infrastructure after the fire. “It’s never been clear,” says Jan, a 20-year resident of Seminole Springs about the park’s emergency preparedness plan. “The [fire] boxes [containing firehoses] we had around the park are dilapidated, they didn’t even work when people ran to use them, they need to do something... but we’re broke.” Since Woolsey, residents have reported that new fire boxes containing hoses have been installed and existing ones have been repaired. Other residents reported being involved in education campaigns within the community, working with nearby fire safe councils to share information about local wildfire risks and develop personal preparedness strategies at the household level. Yet, because rebuilding lost homes and repairing damaged infrastructure has taken up much of the available financial resources, many residents, despite possessing the capabilities to return in rebuilt homes, feel less secure in their capability to prepare for and manage future fire risk in the long-term. “I’m not faulting people for wanting to put it behind them, because it was such a traumatic event for so many people, but everyone has,” says Dana, a 25-year Seminole Springs resident. “It’s going to happen again, and we need to be better prepared and nobody’s going to be because nobody wants to look at it.”

## Discussion

This study finds that voluntary non-migration is an active and ongoing process; communities work collectively to mobilize shared resources to develop their collective capabilities to stay. Many residents believe that the survival of their communities requires first and foremost the development of community self-reliance. Many articulate a belief that their communities cannot solely rely on fire services for protection; rather, they must be prepared to protect themselves through strengthening collective efficacy, or the “social cohesion among neighbors combined with their willingness to intervene on behalf of a common good” (Sampson et al., 1997, p. 918). Evidence of successful property-defenders inspired neighbors, encouraging more residents to plan to resist future evacuation orders and stay behind to defend homes. This adds complexity to previous findings of stability in evacuation decisions, where prior evacuation is argued to predict future evacuation behavior (Thompson et al., 2017), suggesting that the erosion of trust in local institutions compels residents to reconsider when and how to evacuate.

In seeking to harden communities and cultivate preparedness, residents are participating in what social movement scholars term “quiet mobilizations” (Jerolmack & Walker, 2018). Rather than engage in confrontational or contentious strategies to induce changes at the governmental level, residents turn to more conventional forms of social engagement, working with neighbors to build collective efficacy within high-risk communities regardless of local government support. The highly localized nature of such efforts is evident in the language community organizers—targeted strategies like organizing fire brigades far exceed broader calls to combat climate change which is exacerbating fire risk. Preventing wildfire ignition and increasing suppression capabilities are more direct and tangible goals around which residents can mobilize rather than broader climate change mitigation strategies that require greater societal change or force affluent residents to more critically examine their own lifestyles. With the neighborhood as the principal site of intervention, everyday community events become potential vehicles for organizing neighbors. Volunteer fire brigade training sessions bring together residents for a day of equipment training followed by a neighborhood barbecue, blending traditional forms of civic participation with community organizing towards a strategic goal of neighborhood preparedness. Such “hybrid collective events” represent an increasingly prevalent form of collective civic action that fosters and sustains community mobilizations (Sampson et al., 2005, p. 681).

It must be noted that not all communities mobilized in this way after Woolsey. In Seminole Springs, where individual and park-wide financial resources were largely exhausted by rebuilding, few resources remain upon which to build comparably equipped and trained fire brigades. Seminole Springs residents articulated greater degrees of unresolved stress and uncertainty around their continued exposure to wildfire risk. While this finding corroborates research on the prospects for negative consequences of remaining in place on disaster survivors’ well-being (Koslov et al., 2021), it also demonstrates the need to expand our understanding of capabilities to stay beyond just the rebuilding of physical structures. While most interviewees possessed the capabilities to return and rebuild, there was significant variation in the capabilities to prepare for and manage fire-risk and future uncertainty. Residents in the more affluent Malibu communities with greater resources to develop local adaptive capacity (i.e., strengthen their capabilities to prepare and manage fire-risk) expressed feeling more “confident,” “relaxed,” and “at ease,” while Seminole Springs residents, despite articulating a “need to be better prepared,” described feelings of stress and fear when they perceive that “nobody’s going to be.” In effect, Seminole Springs residents lack the “adaptation privilege” that helped their affluent neighbors more effectively re-establish their pre-disaster sense of wellbeing and security (Marino, 2018).

This study is not without limitations. First, as data was primarily collected at the height of the COVID-19 pandemic, there were limited opportunities to engage in in-person observations at the research sites. Second, this relatively small sample was designed to capture the recovery trajectories of homeowners with aspirations to rebuild—other relevant groups, in particular renters, and homeowner’s who never aspired to rebuild, were not included. While some may argue that elite cases of communities like Malibu are exceptional, it must be remembered that approximately one-quarter of all Californians, roughly 11 million people, currently reside in wildland-urban interface communities. This extensive population includes many other middle- to upper- income communities. Beyond Malibu, middle- and upper-class homes are slowly being rebuilt in Ventura and Napa Counties after the Thomas and Tubbs Fires respectively. As more privileged populations in North America and Europe come to experience extreme hazard events with greater frequency, understandings of how residential preferences are formed and pursued in such a context matters not only for predicting likely (im)mobility outcomes, but also for understanding larger shifts climate change adaptation discourses.

## Conclusion

This case supports key findings from the emerging literature on environmental (im)mobilities: When faced with increasing environmental risks, many people develop and pursue explicit non-migration aspirations. Aspirations to stay, rebuild in, and defend communities ravaged by wildfire are largely based on deep attachments to the natural and social worlds residents inhabit. This study also clarifies the concept of capabilities to stay. While the capability to rebuild is largely determined by an individual’s financial resources, insurance, or access to aid made available through disaster policies that prioritize rebuilding in place, the capability to cultivate long-term preparedness was, in this case, largely determined by a community’s ability to collectively mobilize neighborhood financial and social capital. While post-disaster community resilience is, and should be, widely celebrated, pursuing disaster preparedness solely through the empowerment of civic associations risks exacerbating the unequal distribution of vulnerabilities where those capable of investing independently in preparedness prove more capable of living securely in high-risk areas than their neighbors with fewer means.

Future research would benefit from adopting a comparative approach, examining distinct cases of voluntary non-migration to determine when and in what specific material and social contexts people are most capable of staying put in environmentally risky contexts. Understanding who is or is not capable to return, and, by extension, capable to effectively prepare for future shocks, is critical to furthering our knowledge of how environmental disruptions heighten inequality. Lastly, environmental immobility scholarship would benefit more broadly from longitudinal studies examining how (im)mobility aspirations and capabilities continue to evolve among disaster survivors in the years following the extreme event itself, particularly as aspirations to stay come into conflict with diminishing capabilities to stay.

## Data Availability

As part of the informed consent obtained from individual participants in the study was an assurance that interview data (recording, transcripts) would not be publicly distributed so, under the conditions of the project's ethics clearance, I cannot make data available.
